# Construction of H‐Doped PdB Nanocrystals as Electrocatalysts to Modulate Formic Acid Oxidation

**DOI:** 10.1002/advs.202403813

**Published:** 2024-07-09

**Authors:** Huiling Li, Shangqi Zhou, Jiewen Liu, Weibin Wang, Ankang Chen, LiBo Sheng, Jingxiang Zhao, Yan Li, Yongming Sui, Bo Zou

**Affiliations:** ^1^ State Key Laboratory of Superhard Materials College of Physics Jilin University 2699 Qianjin Street Changchun 130012 China; ^2^ Key Laboratory of Photonic and Electronic Bandgap Materials of MOE College of Chemistry and Chemical Engineering Harbin Normal University Harbin 150025 P. R. China

**Keywords:** electrocatalysts, electron transfer, formic acid oxidation reaction, nanocrystals, Pd based hydride

## Abstract

The strong ligand effect in B‐doped Pd‐based (PdB) catalysts renders them a promising anode for constructing formic acid fuel cells (FAFCs) exhibiting high power density and outstanding stability. However, the enhancement of the oxidation barrier is unavoidable in this alloy system owing to the electron transfer (ET) from B to Pd. In this study, a hydrogen doping strategy is employed to open charge freedom in PdB compounds and boost their formic acid oxidation reaction (FAOR) activity by suppressing the ET process. The resulting hydrogen‐doped PdB (PdBH) exhibits an ultrahigh mass activity of up to 1.2A mg^−1^_Pd_, which is 3.23 times that of the PdB catalyst and 9.55 times that of Pd black. Detailed experimental and theoretical studies show that the interstitial hydrogen leads to enhanced orbital hybridization and reduced electron density around Pd. This optimized ligand effect weakens the carbon monoxide adsorption and increases the direct pathway preference of PdBH, resulting in its outstanding catalytic activity for the FAOR. The development of this high‐performance hydrogen‐doped PdB catalyst is an important step toward the construction of advanced light element co‐doped metal catalysts.

## Graphical abstract

Hydrogen doping significantly enhances the activity and stability of PdB‐based formic acid oxidation catalysts. The introduction of hydrogen atoms enhances the ligand effect in PdB alloys and opens up their charge freedom degrees, which reduces the formic acid oxidation barrier and improves the selectivity for direct pathways. Meanwhile, the inherent resistance to carbon monoxide of PdB alloys is maintained during this modifying process.

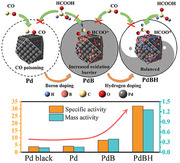

## 1 Introduction

Serious environmental pollution, coupled with the severe fossil fuels shortage, forces mankind to seek innovative, clean, and sustainable energy sources.^[^ ^1^ ^]^ Low‐temperature fuel cells (LTFCs), which can achieve direct and efficient conversion of chemical energy to electricity based on the oxidation of small molecules, including H_2_, CH_3_OH, and CH_4_, are considered the next‐generation energy supply systems in vehicular, portable, and stationary applications.^[^ ^2^ ^]^ As one of the most promising energy conversion systems in LTFCs, direct formic acid fuel cells (DFAFCs) have attracted considerable attention owing to their high theoretical energy density, good storage stability, and low fuel permeability.^[^ ^3^ ^]^ By investigating Pd‐based catalysts for the formic acid oxidation reaction (FAOR), significant achievements have been recently made in assembling DFAFCs with a power density of 550 mW cm^−2^.^[^ ^4^ ^]^ However, owing to the unavoidable carbon monoxide (CO) poisoning of the anode catalysts, the reported DFAFCs have failed to achieve stable operation in practical applications.^[^ ^5^ ^]^ Constructing high‐performance and cost‐effective FAOR catalysts that are resistant to CO is desirable for realizing the commercialization of DFAFCs, but is challenging nonetheless.^[^ ^6^ ^]^

Through strong sp‐d orbital hybridization with Pd, interstitial B doping could effectively reduce the d‐band center energy of Pd crystals, leading to decreased adsorption of CO on the PdB surface.^[^ ^7^ ^]^ Therefore, PdB‐based FAOR catalysts reported so far exhibit outstanding stability, while DFAFCs with a PdB as anode exhibit high power density. Nevertheless, the lower electronegativity of B (2.04) compared to that of Pd (2.20) drives the electron transfer (ET) from B to Pd,^[^ ^8^ ^]^ resulting in an enhanced reaction barrier.^[^ ^9^ ^]^ This increased barrier decreases the catalytic activity of FAOR in PdB systems but is not valued. Considering the higher charge freedom in multi‐doped systems, we suggest that super catalysts with both CO resistance and low reaction barrier can be produced by introducing another element in the PdB systems.

Compared to the widely used metal doping strategies, hydrogen atom doping in the interstitial region can alter the d‐band structure of Pd catalysts to optimise the adsorption of specific reaction intermediates on the catalyst.^[^ ^10^ ^]^ Moreover, the high solubility of hydrogen in Pd and the identical electronegativity make hydrogen a great “diluent” for reducing the ET process in PdB alloy. In this study, we introduced hydrogen atoms into the PdB alloy for the first time and achieved a significant improvement in its catalytic performance. The mass activity of the obtained PdBH catalyst reached 1.2 A mg^−1^_Pd_, which is 3.23 times higher than that of PdB and 9.55 times higher than that of Pd black. The inconsistent shift between the 3d orbital banding energy and d‐band central energy confirms the effectiveness of the hydrogen atoms in regulating the electronic structure of the PdB alloys. Density functional theory (DFT) computations indicated that the synergistic alloying effect of B and hydrogen led to a lower reaction barrier, higher preference for the direct pathway, and weaker adsorption of CO compared to that for PdB, thereby resulting in outstanding catalytic activity and stability.

## 2 Results and Discussion

**Figure 1:**
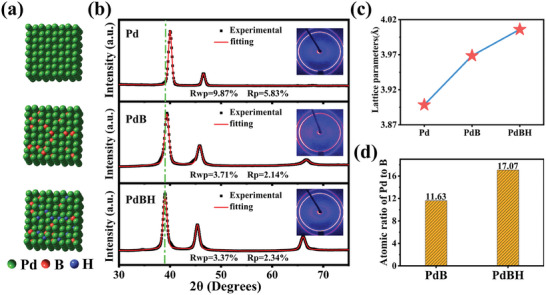
a) Schematic illustration of the formation of PdBH nanocrystals, b) Refined XRD spectra and c) lattice parameters of Pd, PdB and PdBH, d) Atomic ratio of Pd to B in PdB and PdBH. The inset in b) shows the diffraction rings for XRD testing of Pd, PdB and PdBH.

Schematic of the formation of the PdBH nanocrystals is depicted in **Figure** **1**a. Comprehensive synthetic details are provided in the Electronic Supporting Information (ESI). First, Pd nanocrystals without a fixed morphology were synthesised through the reduction of sodium tetrachloropalladium (Na_2_PdCl_4_) by ascorbic acid at 80 °C (Figure S1, Supporting Information),^[^ ^11^ ^]^ following which B was introduced in the lattice interstitial sites of Pd by co‐stirring with NaBH_4_ in a DMF solution at 0 °C.^[^ ^7a^ ^]^ During stirring process, the morphology of the Pd nanoparticles was reconstructed into a cubo‐octahedral morphology under the effect of DMF.^[^ ^12^ ^]^ Nanoparticles with a cubo‐octahedron morphology were obtained under the same conditions even in the absence of NaBH4, confirming that the cubo‐octahedral morphology was related to DMF (Figure S2, Supporting Information). Finally, hydrogen was introduced into the PdB nanocrystals by a hydrothermal method to form PdBH nanocrystals. The X‐ray diffraction patterns (XRD) spectra of the synthesised Pd, PdB and PdBH nanocrystals are shown in Figure 1b. All diffraction peaks of PdB shift toward lower angles relative to those of Pd because of lattice expansion, confirming the interstitial B doping.^[^ ^13^ ^]^ The doping of hydrogen caused further lattice expansion, resulting in the diffraction peak of PdBH continuing to shift toward lower angles.^[^ ^14^ ^]^ The lattice parameters obtained after Pawley refinement of the XRD spectra are shown in Figure 1c, and are 3.898, 3.969, and 4.006 Å for Pd, PdB and PdBH, respectively.

Notably, the Pd/B atomic ratio in PdBH was slightly improved compared to that in PdB, which is related to the successful doping of hydrogen atoms (Figure 1d). To understand the possible effects of hydrogen doping on the morphology of the nanocrystals, scanning electron microscopy (SEM) and transmission electron microscopy (TEM) were conducted.

**Figure 2:**
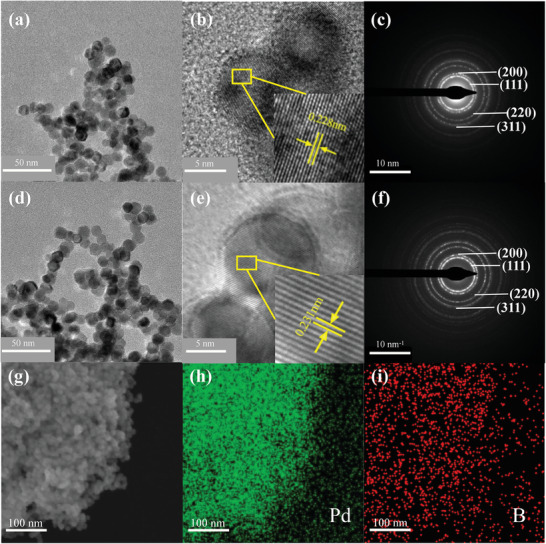
a)TEM, b) HRTEM images and c) SAED pattern of PdB nanocrystals, the inset in (b) shows the interplanar distance of PdB nanocrystals. d)TEM, e) HRTEM images and f) SAED pattern of PdBH nanocrystals, the inset in (e) shows the interplanar distance of PdBH nanocrystals. g–i) SEM and elemental mapping images of PdBH nanocrystals.

Typical TEM images indicate that both PdB and PdBH possess a cubo‐octahedron morphology with particle size distributions of 11.87 ± 1.15 and 11.99 ± 1.09 nm, respectively (**Figure** **2**a,d; Figures S3–S5, Supporting Information). The higher crystallinity of the PdB and PdBH nanocrystals is demonstrated by the resolved lattice fringe of the (111) plane as observed from the high‐resolution TEM (HRTEM) images (Figure 2b,e). The expanded Pd (111) interplanar distance in PdBH was greater than that in PdB owing to hydrogen doping. Selected area electron diffraction (SAED) patterns show four bright concentric rings assignable to the Pd (111), Pd (200), Pd (220), and Pd (311) planes in the PdB and PdBH nanocrystals, which further confirms the invariant space group of Pd after light element doping (Figure 2c,f). SEM and the corresponding elemental mapping images of PdB and PdBH confirmed the uniform distribution of Pd and B in the nanocrystals (Figure 2g–i, Figure S4, Supporting Information).

**Figure 3:**
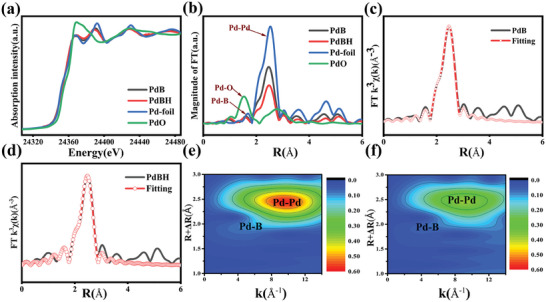
The XANES spectra a) and the Fourier transform of EXAFS spectra b) at the Pd K‐edge, corresponding EXAFS fitting c,d) at R space spectra and e,f) Pd K‐edge wavelet transform contour plots of the PdB and PdBH nanocrystals.

To further investigate the chemical state and coordination environment of the prepared nanocrystals, the Pd K‐edge X‐ray absorption spectra of the PdBH and PdB nanocrystals were recorded and compared with those of PdO and Pd foil. The X‐ray absorption near‐edge structure (XANES) spectra (**Figure** **3**a) of the PdBH and PdB nanocrystals were similar to that of the Pd foil, indicating that Pd was primarily present in a metallic state in these nanocrystals. Fourier transformation of the Pd K‐edge extended X‐ray absorption fine structure (EXAFS) spectra is displayed in Figure 3b. The peak at ≈2.5 and 1.7 Å can be attributed to the Pd─Pd bond and Pd─B bond, respectively, while no peak corresponding to the Pd─O bond (≈1.50 Å) was detected for the synthetic nanocrystals. Moreover, quantitative EXAFS fitting was performed to understand the local chemical coordination environment around the Pd atoms in the prepared nanocrystals (Figure 3c,d; Figures S6–S7, Supporting Information), and the fitting parameters, including the coordination numbers and atomic distances, are listed in Table S1 (Supporting Information).

The Pd─Pd average coordination numbers in the first coordination shell of the Pd foil, PdB, and PdBH are 12, 8.99, and 8.23, respectively; the Pd─B average coordination numbers of PdB and PdBH are 2.05 and 1.53, respectively; and the Pd─Pd average atomic distances in the Pd foil, PdB, and PdBH are 2.73, 2.75, and 2.76 Å, respectively. This result is consistent with the previously discussed XRD and HRTEM data and could be attributed to the interstitial doping of B and hydrogen in the Pd lattice. Wavelet transform contour plots are shown in Figure 3e,f and Figure S8 (Supporting Information); only two intensity maxima were observed at ≈10.2 and 6.7 Å^−1^ for the synthesised nanocrystals, corresponding to Pd─Pd and Pd─B, respectively. The signal of Pd─B is weaker than that of Pd─Pd because of its low coordination number.

**Figure 4:**
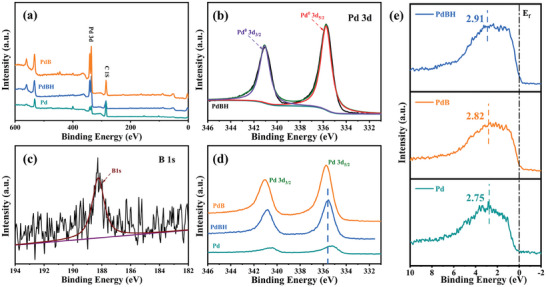
a) XPS survey spectra and b) Pd 3d, c) B 1s regions of the PdBH nanocrystals. comparison of the d) XPS spectra and e) XPS valence band spectra and d‐band centers over the Pd 3d region for PdBH, PdB, and Pd nanocrystals.

X‐ray photoelectron spectroscopy (XPS) was conducted to gain more insight into the electronic structures of Pd, PdB, and PdBH (**Figure** **4**a).^[^ ^15^ ^]^ The high‐resolution XPS spectra of Pd and B for PdB and PdBH are shown in Figure 4b,c and Figures S9 and S10 (Supporting Information). The peaks at ca. 335.7 and 341.0 eV corresponded to metallic Pd 3d_5/2_ and Pd 3d_3/2_ (Figure 4b), while the peak at ≈188.2 eV could be attributed to B^0^ (Figure 4c) in the PdBH nanocrystals.^[^ ^7^, ^16^ ^]^ With the C 1s peak calibrated at 284.8 eV, the core levels of Pd 3d_5/2_ for the PdB nanocrystals show a positive shift relative to those of the initial Pd crystals, with the peak position shifting from 335.34 to 335.74 eV (Figure 4d; Table S2, Supporting Information). This shift was attributed to the ET from B to Pd.^[^ ^17^ ^]^ This direction of ET is consistent with the electronegativity (2.20 for Pd and 2.04 for B) and work function (5.22 eV for Pd and 4.98 eV for B).^[^ ^8^ ^]^ The positive shift of 1s core level peak of B in PdB (188.29 eV) relative to the peak of B powder (187.91 eV) also indicates the occurrence of ET (Figure S11 and Table S2, Supporting Information). The resulting electron‐enriched Pd surface lowers the work function of the PdB alloy, which is obviously unfavourable for the oxidation reaction.^[^ ^18^ ^]^ Furthermore, as expected, the Pd 3d_5/2_ core level peak of PdBH (335.60 eV) exhibits a significant negative shift compared to that of PdB(335.74 eV), while the core levels of B 1s barely changed (Figure 4d; Figure S11 and Table S2, Supporting Information). This confirms that hydrogen doping is effective in reducing the electron density on the PdB surface. In general, core‐level shifts are a good indicator. (“fingerprint”) of the shift in the center of the occupied d‐states for elements with almost filled d‐bands.^[^ ^19^ ^]^ However, in our case, the valence level of PdBH exhibited an unexpected down‐shift relative to that of PdB according to the XPS valence band spectra (Figure 4e). The center of the platinum‐group metals valence band is determined by their ET and orbital hybridization behavior with alloying elements.^[^ ^20^ ^]^ Hydrogen doping can reduce the electron density on the surface of Pd, thus reducing the Fermi level. On the other hand, strong s‐d orbital hybridization affords significant ligand effects, resulting in a down‐shift in the center of the valence level.^[^ ^10b^ ^]^ Evidently, in this work, orbital hybridization dominates the central shift of the valence band in the PdBH nanocrystals.

Further, we characterised the surface electronic structures of the Pd, PdB, and PdBH nanocrystals from the high‐resolution XPS core‐level spectra and valence band spectra. The results indicate that electronic adjustments and orbital coupling of hydrogen atoms on the surface of the PdB and PdBH nanocrystals reduce the surface electron density and valence band level, which are beneficial for promoting the oxidation process and reducing the adsorption of oxygen‐containing species. The higher work function (Figure S12, Supporting Information) of the PdBH nanocrystals further confirms our conclusion. Based on this, we believe that PdBH has more efficient and stable catalytic performance toward the FAOR. Therefore, we further conducted electrochemical tests on the Pd, PdB, and PdBH nanocrystals.

**Figure 5:**
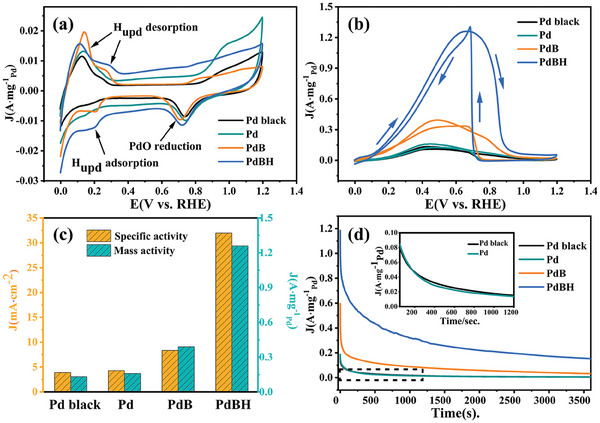
The electrochemical measurements of Pd black, Pd, PdB, and PdBH catalysts a) CV in 0.5 m H_2_SO_4_ at a scan rate of 50 mV s^−1^ b) Mass‐normalized CV curves of the catalysts in a 0.5 m H_2_SO_4_ solution containing 0.5 m HCOOH. c) Comparisons of mass activity and specific activity. d) Chronoamperometric curves for 3600 s. The inset in (d) shows the localized magnifications of Pd catalysts and Pd black.

Formic acid electrooxidation was evaluated as a model reaction to investigate the electrocatalytic performance of the PdBH, PdB, Pd nanocrystals, and Pd black. First, cyclic voltammetry (CV) measurements were carried out in 0.5 m H_2_SO_4_ with N_2_ saturation at a scan rate of 50 mV s^−1^ to investigate the adsorption/desorption and redox characteristics of the catalysts in electrochemical environments. All potentials were converted to values for the reference reversible hydrogen electrode (RHE). In **Figure** **5**a, the distinct peaks in the region from 0 to 0.3 V are referred to as the underpotential adsorption and desorption of H. Additionally, there is a noticeable decrease in the peak associated with the reduction of Pd oxide species in the range of ≈0.6–0.9 V.^[^ ^21^ ^]^ Owing to hydrogen adsorption on Pd surfaces, electrochemical surface area (ECSA) measurements via hydrogen underpotential deposition would be inaccurate. Therefore, ECSAs were calculated from the Pd redox peaks. Details of the calculation method are provided in the electronic supporting information. The ECSA values of PdBH, PdB, Pd, and Pd black were 3.939, 4.711, 3.692, and 3.392 m^2^ g^−1^, respectively. The results indicate that the doping of light elements increases the ECSA of the Pd‐based catalysts, which is related to the increase in the number of active sites (Figure S13, Supporting Information).

Furthermore, the electrocatalytic activities of these four catalysts for the FAOR were assessed from the CV curves obtained in N_2_‐saturated 0.5 m H_2_SO_4_+0.5 m HCOOH at a scan rate of 50 mV s^−1^. Forward scanning of all CV curves began at 0.003 V versus RHE (Figure 5b). The first peak in the forward scanning curve is attributed to the formic acid oxidation accompanied by CO_2_ release (direct oxidation: HCOOH → CO_2_ + 2H^+^ +  2e^−^). The second peak was attributed to the indirect oxidation (HCOOH → CO_ads_ + H_2_O → CO_2_ + 2H^+^ + 2e^−^). The peak at about 0.6 to 0.8 V in the reverse scan was attributed to Pd oxide reduction, which was accompanied by recovery of the active sites of the catalyst.^[^ ^22^ ^]^ In contrast to those of PdB, Pd, and Pd black catalysts, no significant indirect oxidation pathway peak appeared in the CV curves of PdBH, suggesting its increased preference for the direct pathway due to hydrogen doping. Moreover, the current density of the PdBH catalyst is much higher than that of the other catalysts. For a quantitative comparison, specific activity and mass activity are obtained by normalising the CV current with respect to the ECSA and total Pd loaded on the working electrode (Figure 5c; Figure S14, Supporting Information). The Pd contents in the catalysts were determined by ICP‐MS (Table S3, Supporting Information). The mass activity of PdBH reaches 1.260 A mg^−1^_Pd_, which is 3.23, 7.97 and 9.55 times higher than those of PdB (0.390 A mg^−1^_Pd_), Pd (0.158 A mg^−1^_Pd_), and Pd black (0.132 A mg^−1^_Pd_), respectively. The specific activity of PdBH is 31.991 mA cm^−2^, which is 3.83, 7.47 and 8.22 times higher than those of PdB (8.349 mA cm^−2^), Pd (4.284 mA cm^−2^), and Pd black (3.894 mA cm^−2^), respectively. Obviously, the addition of hydrogen improved the oxidation activity of the PdB catalyst significantly, establishing it as one of the best Pd‐based FAOR electrocatalysts reported so far (Table S4, Supporting Information).

The stability of the electrocatalysts is one of the most critical parameters to determine the commercial application of the FAOR.^[^ ^23^ ^]^ Chronoamperometry (CA) was performed for 3600 s to further assess the electrochemical stability of electrocatalysts. As seen in Figure 5d, the electrooxidation of formic acid and the reduction in current density were rapid in the initial stage and eventually stabilised at a fixed value. This is due to the double‐layer discharge and the adsorption and accumulation of reaction intermediates (such as ^*^CO) on the surface of the active sites. PdBH showed the slowest current decay rate among all the catalysts and retained a mass activity of ≈0.2 A  mg^−1^_Pd_ after 3600 s of CA testing, demonstrating its outstanding catalytic stability in the FAOR. Figures S15–S18 (Supporting Information) shows the TEM images and XRD patterns of the synthesised catalyst before and after 3600 s of CA testing. It is evident that the morphology and XRD patterns of PdBH, PdB, and Pd did not change significantly after the FAOR testing, indicating their structural stability. Furthermore, CO stripping measurements (Figure S19, Supporting Information) indicated the lowest CO desorption energy of PdBH, suggesting weak CO adsorption owing to the downward shift of the d‐band centers due to the ligand effects. In summary, electrochemical tests show that PdBH has excellent catalytic activity and resistance to CO, which supports our XPS analysis. DFT calculations were conducted to further clarify the origin of this excellent catalytic performance.

**Figure 6:**
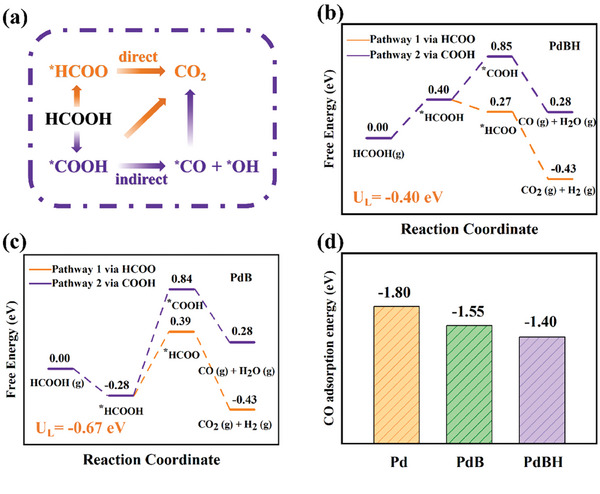
a) Simplified reaction pathways of electrocatalytic FAOR. Comparison of the free energy profiles of PdBH b) and PdB c) catalysts via direct and indirect pathways. d) Adsorption energy of ^*^CO on Pd, PdB, and PdBH catalysts.

To gain deeper insights into the superior catalytic performance of the PdBH catalysts for formic acid oxidation, we conducted grand‐canonical DFT (GC‐DFT) computations. The catalytic performance of these Pd‐based catalysts for formic acid oxidation was evaluated by computing the free energy changes of all elementary steps. Two reaction mechanisms for formic acid oxidation including direct and indirect mechanisms were considered in this work (**Figure** **6**a).

The results suggested that HCOOH was first adsorbed on the PdBH catalysts by forming one Pd─O bond with a length of 2.25 Å. Remarkably, E_ads_ for HCOOH adsorption was computed to be −0.36 eV, corresponding to a ∆G of 0.40 eV after considering the contributions from the zero‐point energy and entropy. Subsequently, the adsorbed HCOOH species were dehydrogenated to ^*^HCOO or ^*^COOH species by the dissociation of its O─H or C─H bond. A comparison of the free energy of each step revealed that a small energy input of 0.40 eV was required for hydrogen‐doped PdB during the entire reaction (Figure 6b), while the energy input to initiate the entire formic acid oxidation was as high as 0.56 and 0.67 eV for pristine Pd (Figure S20, Supporting Information) and B‐doped Pd catalysts (Figure 6c), respectively. Obviously, the incorporation of hydrogen reduced the energy input required for the entire reaction, which was conducive to the FAOR. Furthermore, the ^*^HCOO formation was exergonic for PdBH, with a ∆G value of −0.13 eV, whereas it is endergonic for PdB (∆G = 0.67 eV). Considering that ^*^COOH formation is endergonic for both PdB and PdBH, we suggested that PdBH had a higher selectivity for the direct path. During the FAOR process, CO poisoning must be avoided, as the formed CO may block the catalyst surface, thus impeding the further oxidation of formic acid. To this end, we computed the adsorption strength of the CO species on the pristine Pd, PdB, and PdBH catalysts (Figure 6d). The computed E_ads_ values for the three catalysts were −1.80, −1.55, and −1.40 eV, respectively, suggesting that the introduction of B, and especially the BH dopant, could relieve the CO poisoning to some extent, which normally helped to boost the FAOR. This result was consistent with the CO stripping experiment. According to the above discussion, hydrogen doping reduced the energy input to the FAOR and the adsorption strength of the CO species, in addition to providing PdB with greater selectivity toward the direct mechanism, resulting in significant improvement in the FAOR activity of the PdBH catalyst.

## 3 Conclusion

In conclusion, we introduced hydrogen into the PdB system through simple hydrothermal treatment. As obtained PdBH catalyst exhibited a superior mass activity of 1.2 A mg^−1^_Pd_, which is 3.23 and 9.55 times higher than those of PdB and Pd black, respectively. Moreover, PdBH retained a mass activity of ≈0.2 A mg^−1^_Pd_ after 3600 s of CA testing, while PdB and Pd black were completely deactivated under these conditions. Detailed experimental and theoretical studies showed that hydrogen enhanced the ligand effect in PdB and reduced the electron density around Pd. Therefore, the PdBH nanocrystals exhibited low FAOR barrier, high preference for the direct pathway, and excellent resistance to CO, leading to its outstanding catalytic activity for the FAOR. This work demonstrates the effectiveness of the hydrogen doping strategy to obtain efficient PdB‐based FAOR catalysts and provides a novel perspective for the design of emerging light element co‐doped metal catalysts.

## Conflict of Interest

The authors declare no conflict of interest.

## Supporting information

Supporting Information

## Data Availability

The data that support the findings of this study are available from the corresponding author upon reasonable request.
