# Supplementary material for: Construction of H‐Doped PdB Nanocrystals as Electrocatalysts to Modulate Formic Acid Oxidation
Source: Adv Sci (Weinh). 2024 Jul 9;11(34):2403813. doi: 10.1002/advs.202403813 (PMC11425968; doi:10.1002/advs.202403813)
Supplement: Supplementary file 1 — Supporting Information [file ADVS-11-2403813-s001.docx]

Supporting Information
©Wiley-VCH 2021
69451 Weinheim, Germany

**Construction of H-doped PdB Nanocrystals as Electrocatalysts to Modulate Formic Acid Oxidation**

Huiling Li, ^[1]^ Shangqi Zhou, ^[2]^ Jiewen Liu, ^[1]^ Weibin Wang, ^[1]^ Ankang Chen, ^[1]^ LiBo Sheng, ^[1]^ Jingxiang Zhao, * ^[2]^ Yan Li,*^[1]^ Yongming Sui* ^[1]^ and Bo Zou ^[1]^

Dedication: Huiling Li and Shangqi Zhou contributed equally to this work.

[1] H. Li, J. Liu, W. Wang, A. Chen, L. Sheng, Prof. Y. Li, Prof. Y. Sui, Prof. B Zou
State Key Laboratory of Superhard Materials
College of Physics, Jilin University
Changchun 130012, China 2699 Qianjin Street
E-mail:liyan2012@jlu.edu.cn;suiym@jlu.edu.cn

[2] S. Zhou, Prof. J. Zhao
Key Laboratory of Photonic and Electronic Bandgap Materials of MOE
College of Chemistry and Chemical Engineering, Harbin Normal University
Harbin, 150025, PR China

E-mail: zhaojingxiang@hrbnu.edu.cn

Experimental

- 1. Chemicals and Materials.

Na_2_PdCl_4_, (99.99%), ascorbic acid (99.99%), Pd black (99.95%) Isopropyl Alcohol (99.9%), Formic acid (88%) were purchased from aladdin. N,N-Dimethylformamide(99.5%), Acetone（99.5%）, PVP (Mw ≈ 55,000), KCl, NaBH_4,_ Nafion solution (5%)were purchased from Titan. All the chemicals were used as received without further purification. All aqueous solutions were prepared using Milli-Q water with a resistivity of 18.2 MΩ·cm at room temperature.

- 1. Synthesis of Pd nanocrystals without fixed morphology.

Palladium nanocrystals without fixed morphology were synthesized by modifying the synthesis method reported by Xia, ^[1]^80 mL of deionized water containing 1.05 g of polyvinyl pyrrolidone(PVP , MW ~55000), 600 mg of L-ascorbic acid (AA) and 1.85 g of KCl were dissolved in a 150 ml vial and heated in an oil bath under magnetic stirring at 80 °C for 10 min. Afterwards, 30 mL of an deionized water solution containing 570 mg of Na_2_PdCl_4_ was injected, the reaction solution was kept at 80 °C for 3 h. The Pd nanocrystals without fixed morphology was collected by centrifugation and washed with water and acetone for several times.

- 1. Synthesis of PdB nanocrystals with cubo-octahedron morphology.

For boron doping, referring to Wang's method,^[2]^ the obtained Pd nanocrystals without fixed morphology (30 mg) were dispersed in DMF (10 mL) under ultrasonication, followed by addition of DMF solution (1mL) containing NaBH_4_ (25 mg) with vigorous stirring for 2 h in an ice-cooled water bath. After centrifugation, washing, and drying, the PdB nanocrystals with cubo-octahedron morphology were obtained.

- 1. Synthesis of PdBH nanocrystals with truncated octahedral morphology.

10 mg prepared PdB nanocrystals with cubo-octahedron morphology were dispersed in 20 mL of DMF. This combination was poured into a Teflon-lined stainless-steel autoclave. The sealed vessel was heated from room temperature to 180°C in around 60 min and kept at this temperature for 2 h then allowed to cool to room temperature naturally. The product was obtained by subsequent centrifugation at 10000 rpm 5 min and then washed with ethanol 3 times and dried at room temperature.

- 1. Characterization.

The phase structure of all samples was characterized by a Rigaku X-ray diffraction (XRD, R-AXIS-RAPIDII, Cu K α radiation, λ =1.5406 Å). The morphology, elemental and crystal characteristics of the samples were identified by scanning electron microscopy(SEM, Regulus 8100, Hitachi) and transmission electron microscopy (TEM, JEM-2100F, JEOL). The surface composition and metal values of samples were analyzed by X-ray photoelectron spectroscopy (XPS, ESCALAB-250Xi). The contents of Pd and B were determined by inductively coupled plasma mass spectrometry (ICP-MS, iCAP, RQ).

- 1. Electrochemical measurements.

First, 5mg Vulcan XC-72R carbon black and 1mg Pd catalyst sample were dispersed in 1ml solution containing 0.5ml ethanol and 0.5ml deionized water, and then added to 10ul nafion ice bath for 1h ultrasonic treatment. Obtain a well-mixed catalyst/C inks and the content of Pd in catalyst was determined by ICP-MS. Then 2.5ul the catalyst/C ink was deposited on a pre-cleaned glassy-carbon electrode with a diameter of 3 mm and dried at ambient condition. Electrochemical measurements are carried out in a standard three-electrode cell system using an electrochemical workstation (CHI 660E, CH Instrument, Inc.) A glassy carbon electrode (GCE, 3mm) with a catalyst coating serves as the working electrode. The Ag/AgCl electrode served as the reference electrode and the Pt plate as the counter electrode. According to E_(RHE)_ = E_(Ag/AgCl)_ + 0.197 + 0.059 ×pH, all potentials were converted to values for the reference reversible hydrogen electrode (RHE). To guarantee a clean surface before use，the GCE was polished by Al_2_O_3_ slurry with particle sizes of 300 and 50 nm, respectively, followed by washing with Milli-Q water and ethanol then the catalyst ink is dropped on its surface and dried at room temperature. The Cyclic voltammogram (CV) were measured in N_2_-saturated 0.5M H_2_SO_4_ solutions and N_2_-saturated solutions containing 0.5M H_2_SO_4_ and 0.5M HCOOH, respectively, with a potential range from -0.2 V to1 V (Vs. Ag/AgCl) at a scan rate of 50 mV∙ s^-1^

The electrochemically active surface areas (ECSA) were calculated based on the charge required for oxygen desorption, that is, from the area of the reduction peak of PdO in the as-obtained CV curves. According to the equation ECSA = Q/ 0.420 m estimate the ECSA values. In this equation, m denotes the Pd mass on the working electrode surfaces. Q value represents the reduction charge of PdO. 0.420 mC cm^−2^ is a constant assuming that a monolayer of PdO is reduced on Pd surface. ^[3]^

- 1. Statistical Analysis

In our work, all of the nanocrystal sizes were measured using the following method. Firstly, TEM images of NCs were obtained with a JEM-2200FS with an emission gun operating at 200 kV. Secondly, the TEM images were introduced into the Nano Measurer 1.2 software and the size of the nanocrystals were calibrated. Thirdly, the calibrated NCs size data were introduced into the Origin 2018 software and fitted with Gaussian Function to obtain the average crystallite size of the NCs. The measurement data were finally represented as the mean ± standard error (SE).

- 1. Computational models and methods.

All spin-polarized density functional theory (DFT) computations were carried out by using the Vienna Ab Initio Simulation Package (VASP) with the projector-augmented wave (PAW) method and a cutoff energy of 400 eV was adopted. The exchange–correlation interactions were described by the Perdew–Burke–Ernzerhof (PBE) functional within the generalized gradient approximation (GGA). The convergence thresholds for energy and force were set to 10^−4^ eV and 0.05 eV Å^−1^, respectively. The Pd (111) surface was simulated with a 4×4 four-layer slab with the top two layers relaxed and the bottom two layers fixed in all calculations, in which a vacuum space of 20 Å in the z-direction was adopted to avoid the interactions between periodic images. Notably, according to our computations, the B atom prefers to be inserted the Pd sublayer, whereas H dopant is energetically favorable on the Pd surface (Figure S16-S19), which were thus chosen as the catalysts for formic acid oxidation. The Brillouin zone was sampled with a Monkhorst–Pack grid of 3 × 3 × 1 for geometry optimization.

The adsorption energy (E_ads_) of CO molecule on Pd-based catalysts was defined as: E_ads_ = E_CO*_ - E_Pd_ -E_CO_, where E_CO*_, E_Pd_, and E_CO_ represent the total electronic energies of the adsorbed CO species, the pristine Pd-based catalysts, and the isolated CO molecule, respectively. The Gibbs free energy change (∆G) for each elementary step for the FAOR process was determined according to the computational hydrogen electrode (CHE) model: ∆G = ∆E +∆ZPE -T∆S +∆G_pH_ + eU $\Delta G = \Delta E + \Delta ZPE - T\Delta S + {\Delta G}_{\mathrm{pH}} + eU$, where ∆E is the reaction energy of the reactants and products adsorbed on the catalyst, calculated directly by DFT; ∆ZPE and ∆S are the changes in zero point energy and entropy at 298.15 K, which can be calculated by the vibration frequency. ∆G_pH_ is the free energy correction of pH, which can be calculated by: ∆G_pH_=K_B_T×pH×ln10. Furthermore, to simulate the realistic electrochemical system, the constant potential model (CPM) was employed by performing the grand canonical DFT (GC-DFT) computations.

2.Supplementary figures and tables


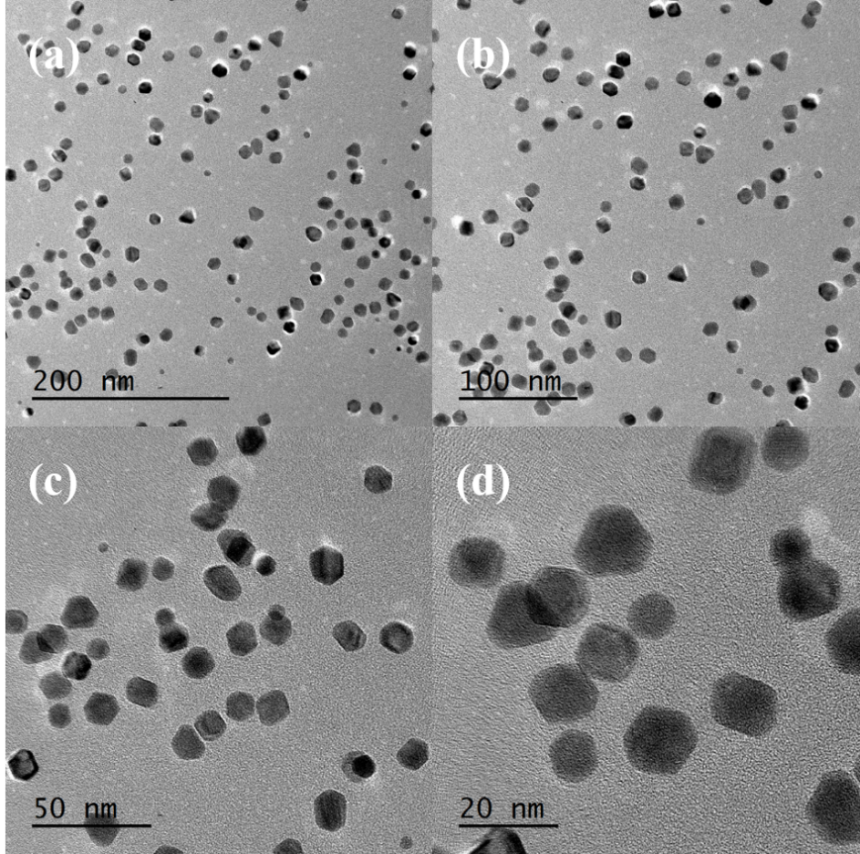


**Figure S1.** TEM images with different magnifications of Pd nanocrystals


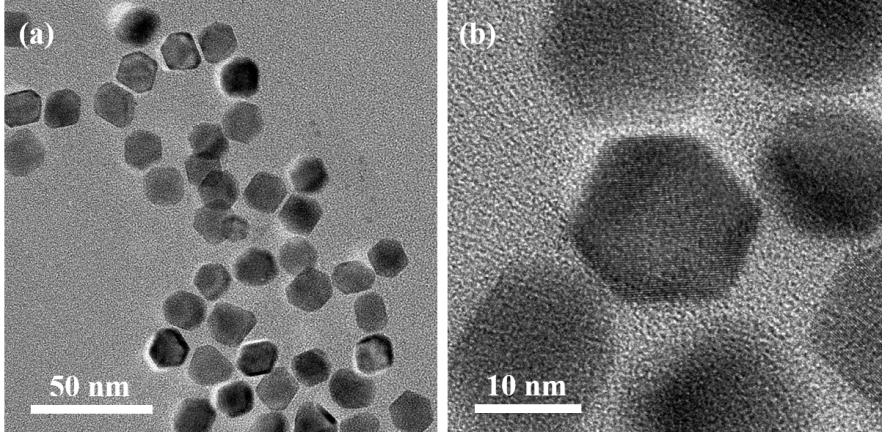


**Figure S2.** TEM image of nanoparticles obtained by removing NaBH_4_ and placing Pd nanocrystals in DMF in an ice water bath for 2 hours


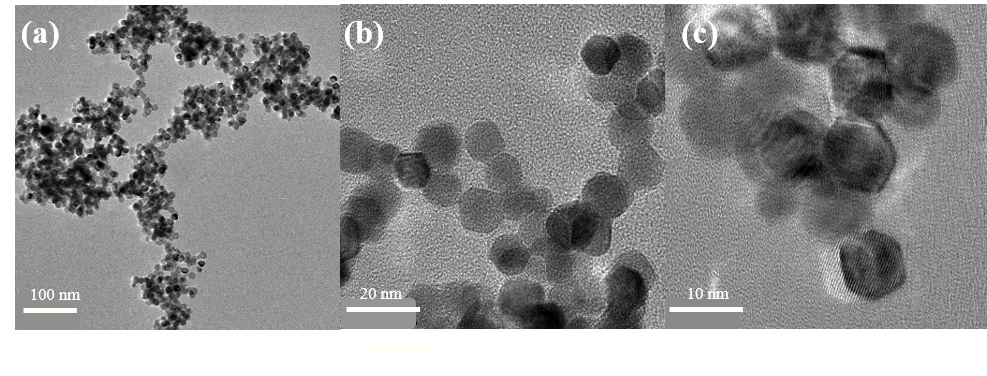


**Figure S3.** TEM images with different magnifications of PdBH nanocrystals


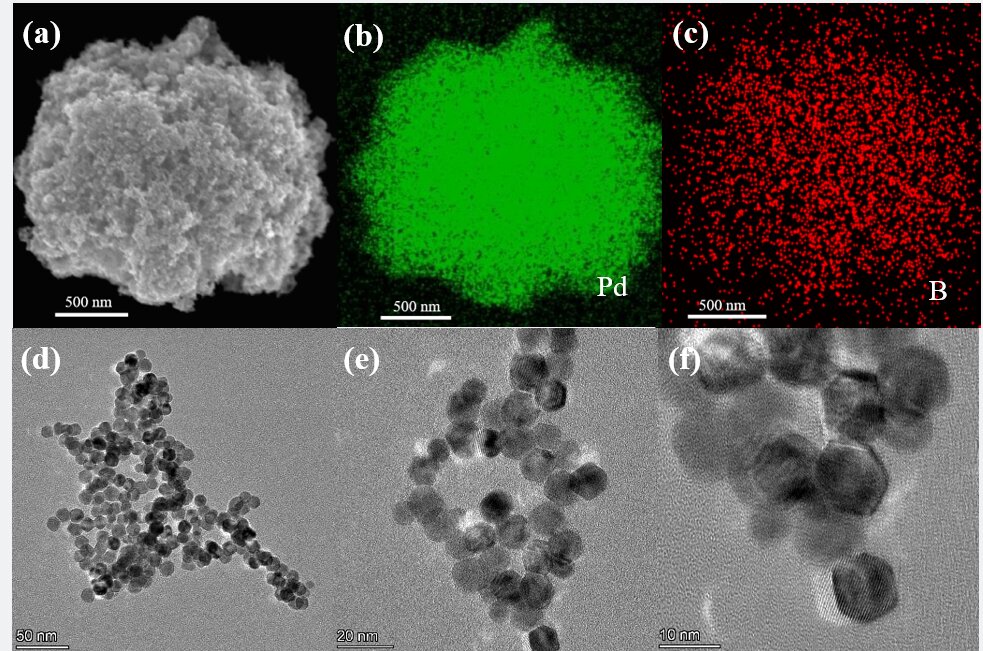


**Figure S4.** Electron microscopy characterization. (a)-(c) SEM and elemental mapping images , (d)-(g) TEM images with different magnifications of PdB nanocrystals


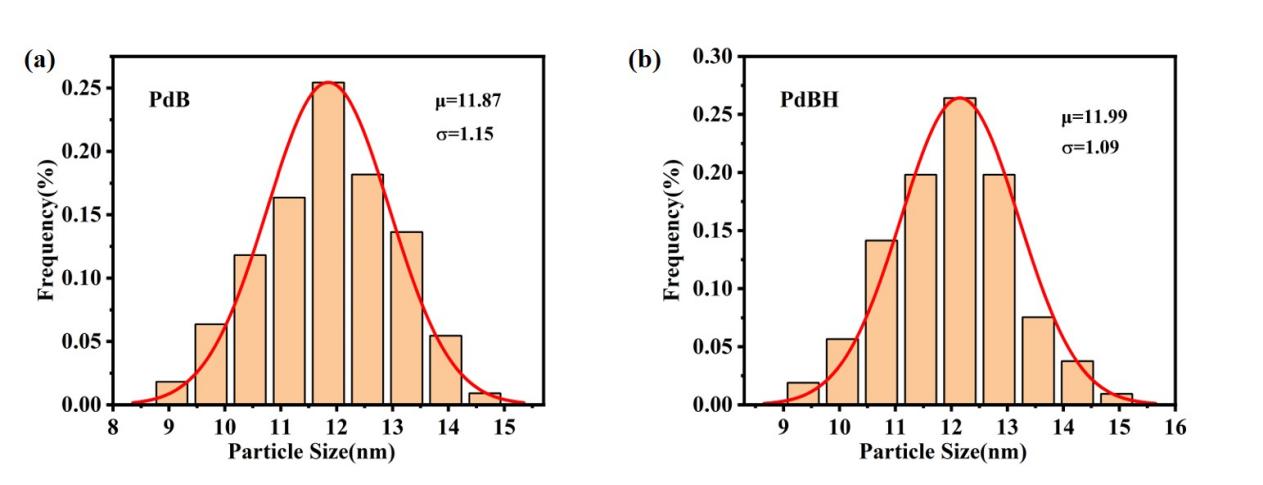


**Figure S5.** Particle size distribution of the PdB (a) and PdBH (b) nanocrystal


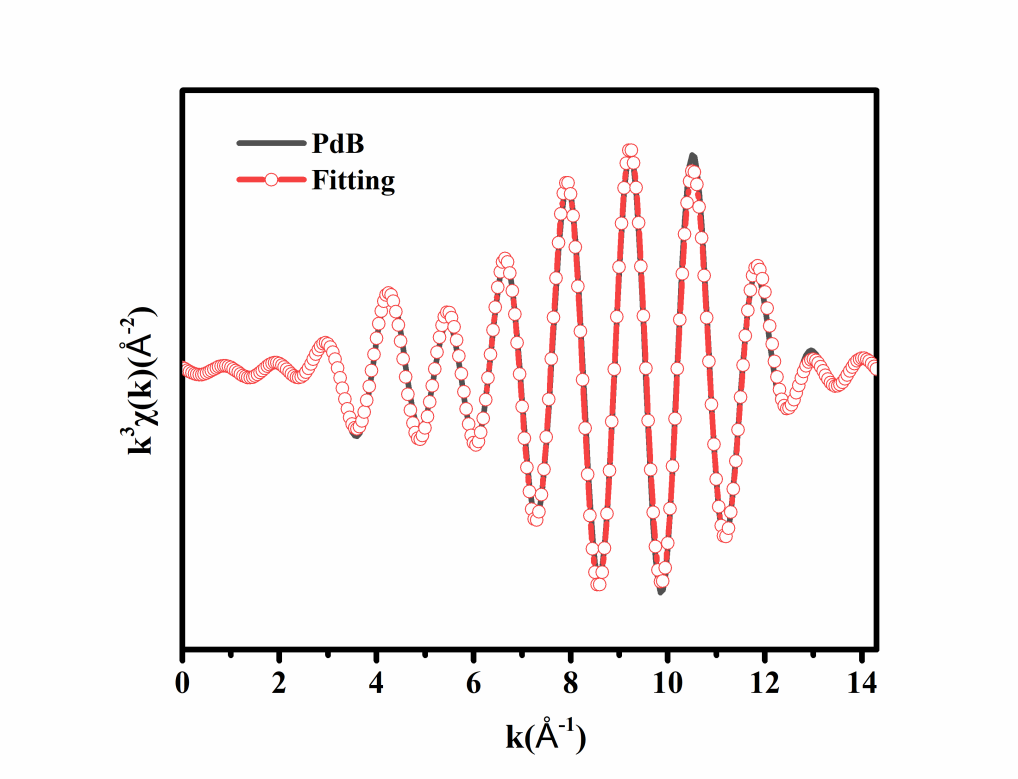


**Figure S6.** The corresponding EXAFS fitting curves of the PdB catalyst at k space.


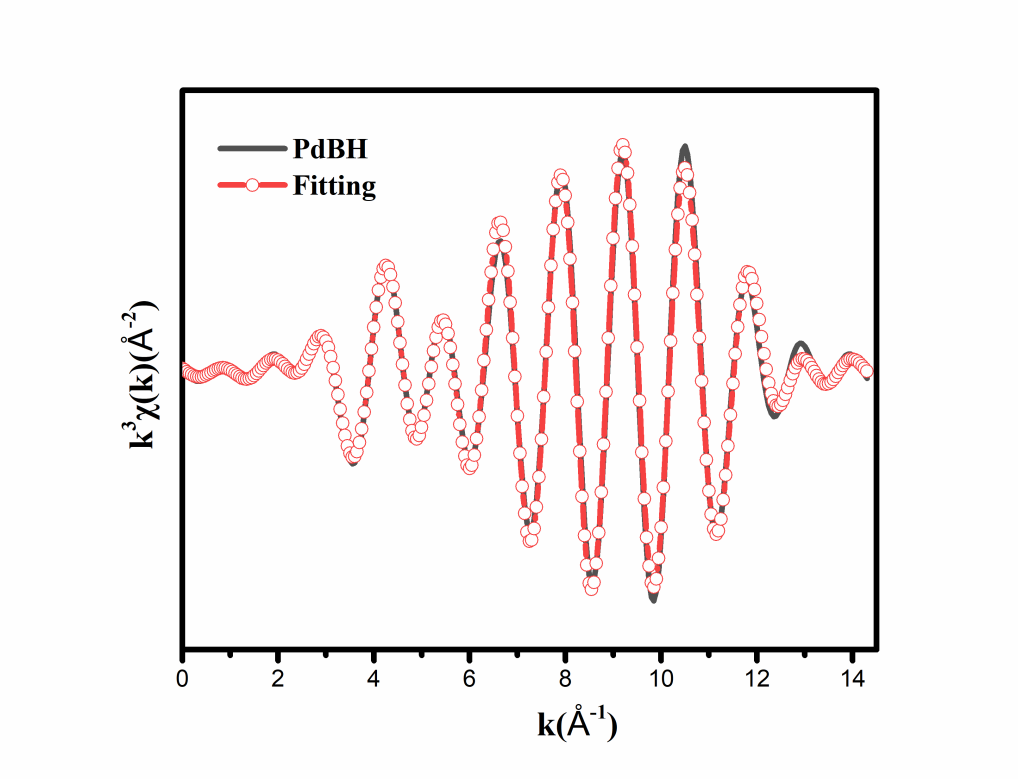


**Figure S7.** The corresponding EXAFS fitting curves of the PdBH catalyst at k space.


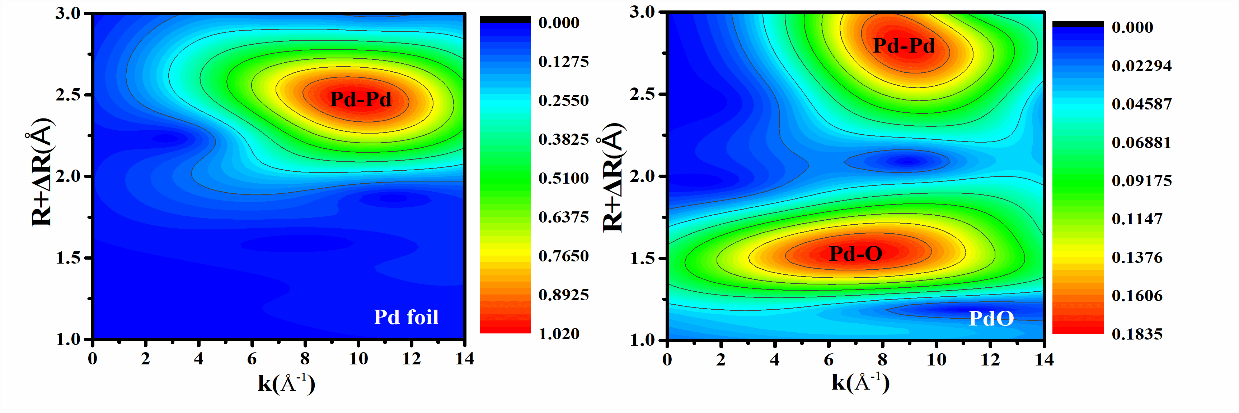


**Figure S8.** Pd K-edge wavelet transform contour plots of Pd foil and PdO.


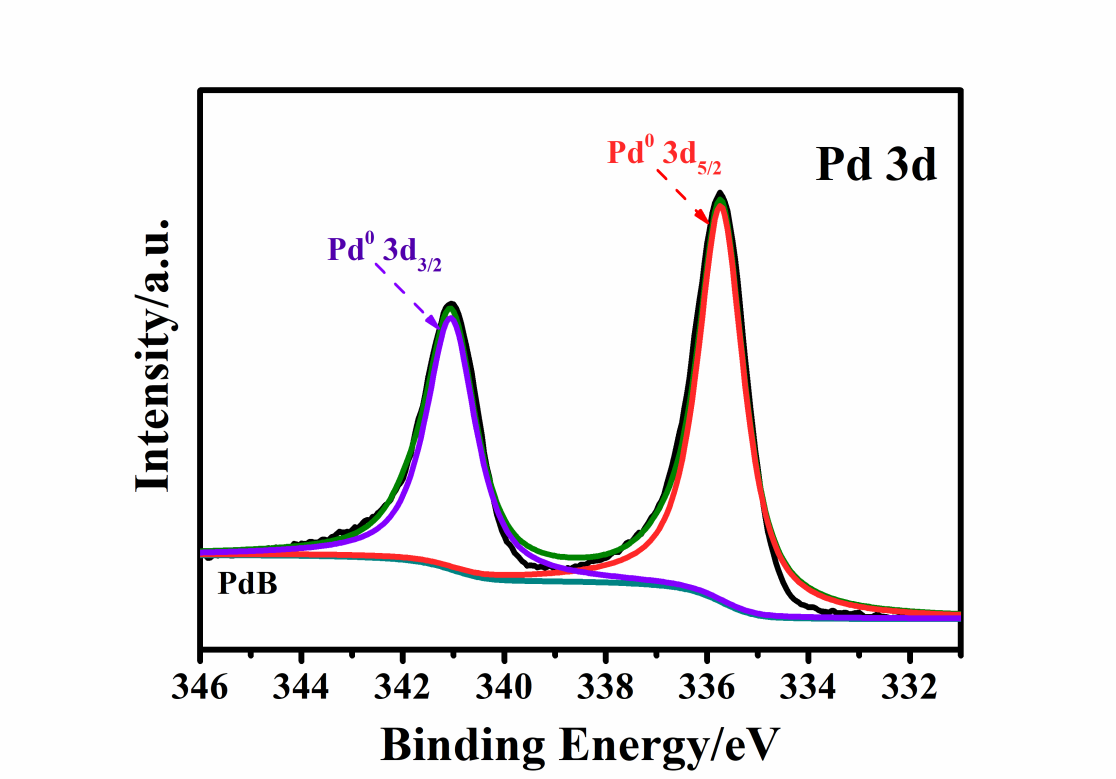


**Figure S9.** XPS spectra of the Pd 3d region for the PdB nanocrystals.


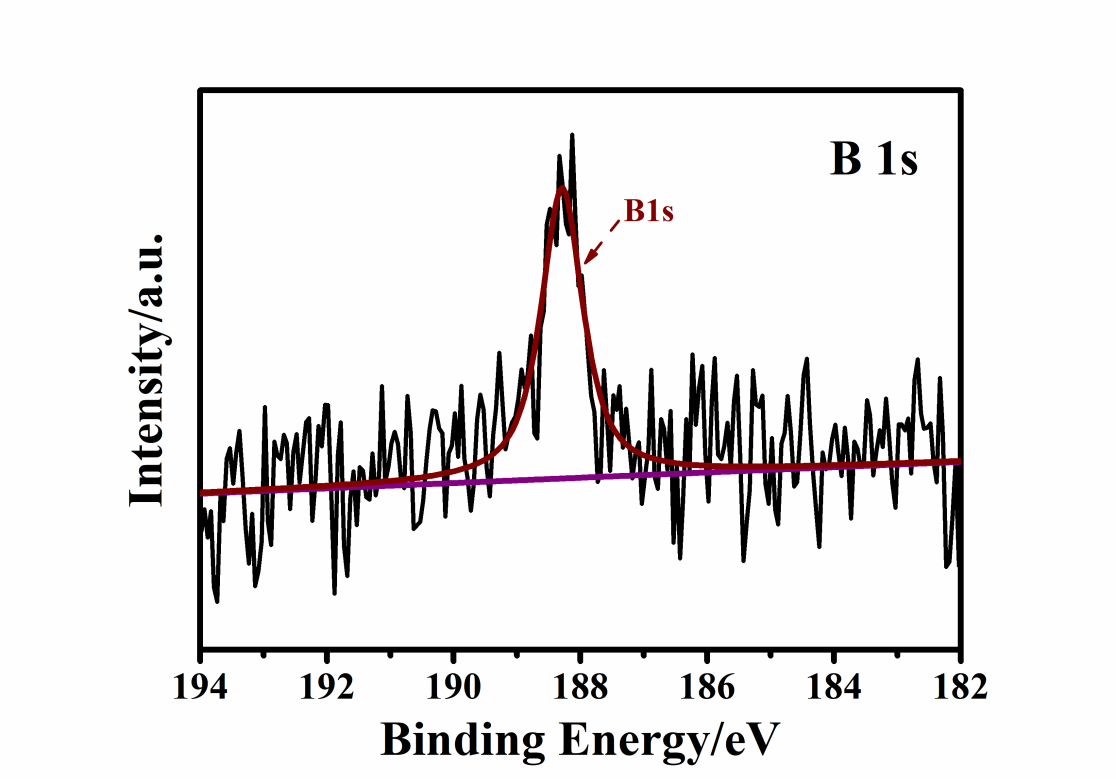


**Figure S10.** XPS spectra of the B 1s region for the PdB nanocrystals.


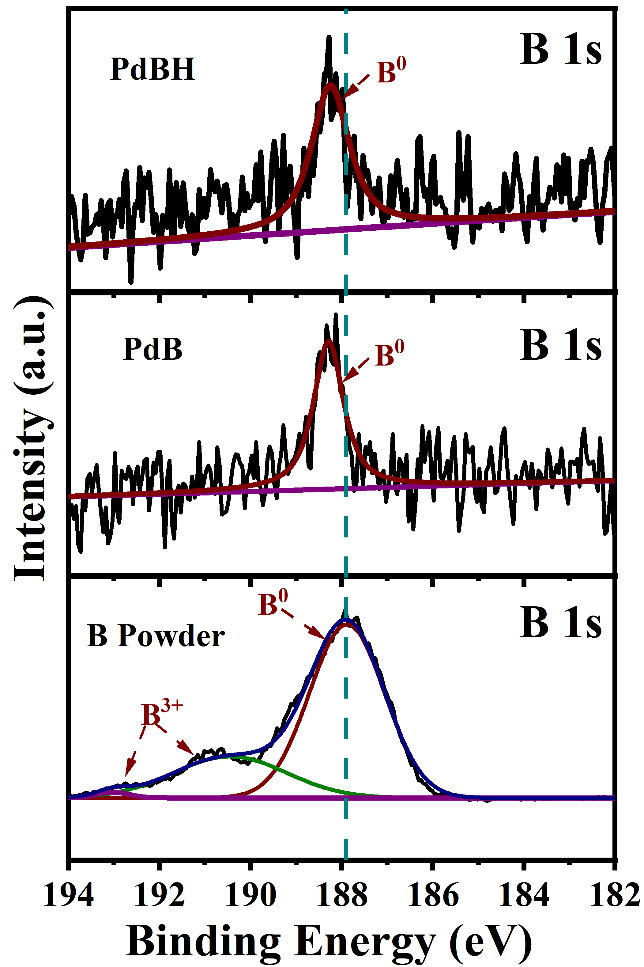


**Figure S11.** Comparison of the XPS spectra of B 1s region for PdBH, PdB nanocrystals and B powder.


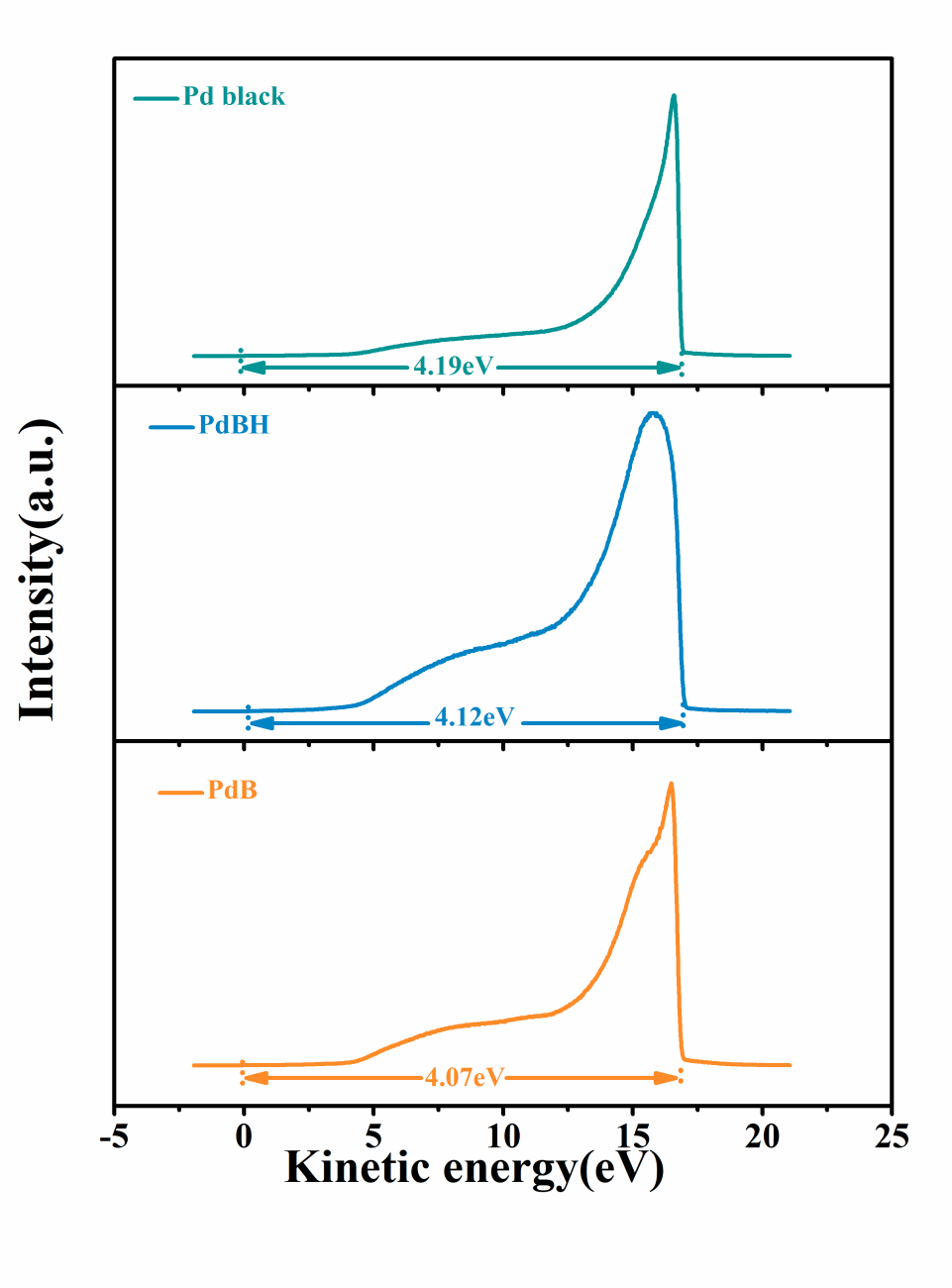


**Figure S12.** UPS spectra and work function of the PdBH, PdB nanocrystals and Pd black.


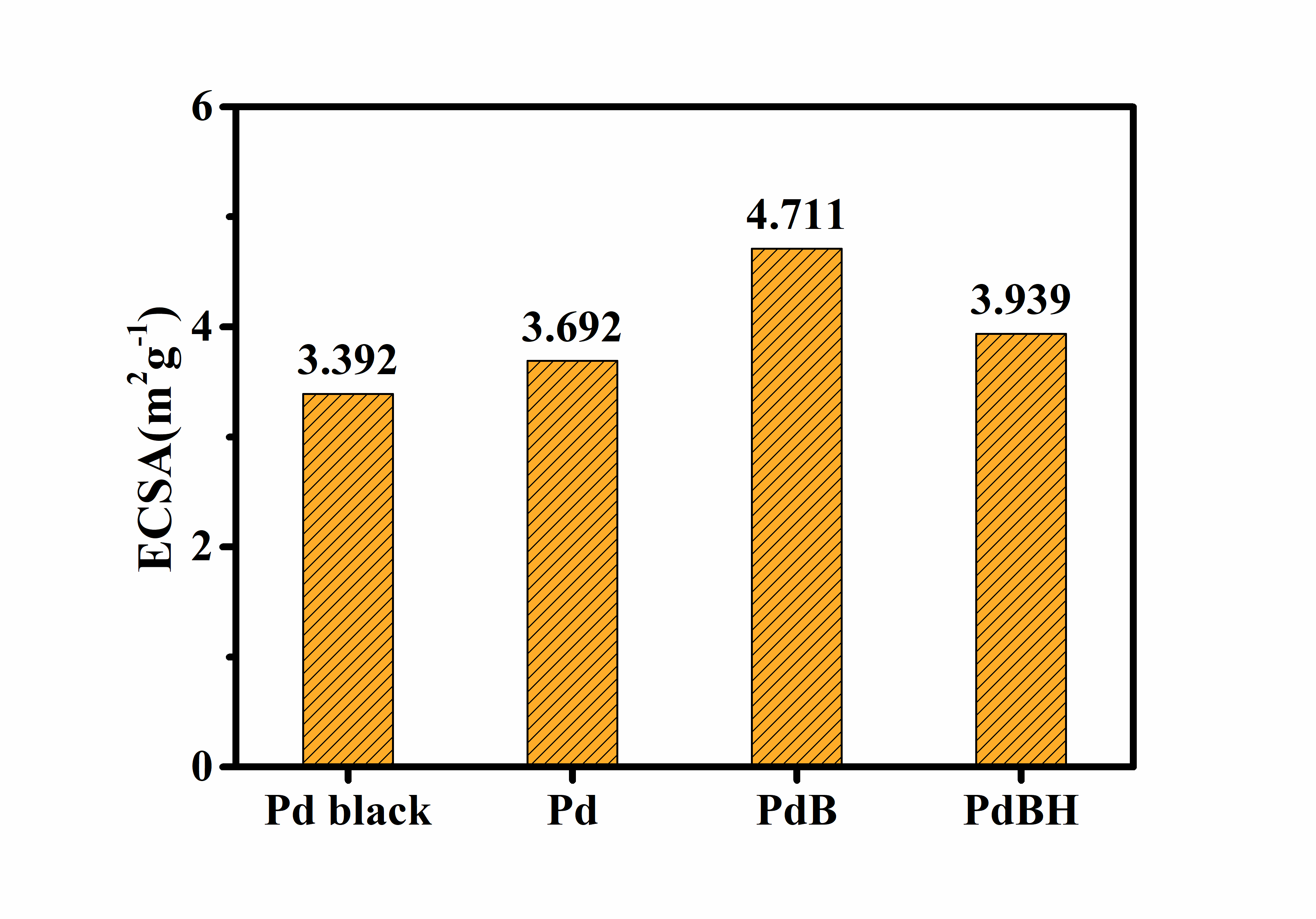


**Figure S13.** Comparisons the electrochemical surface area (ECSA) of Pd black, Pd, PdB, and PdBH catalysts.


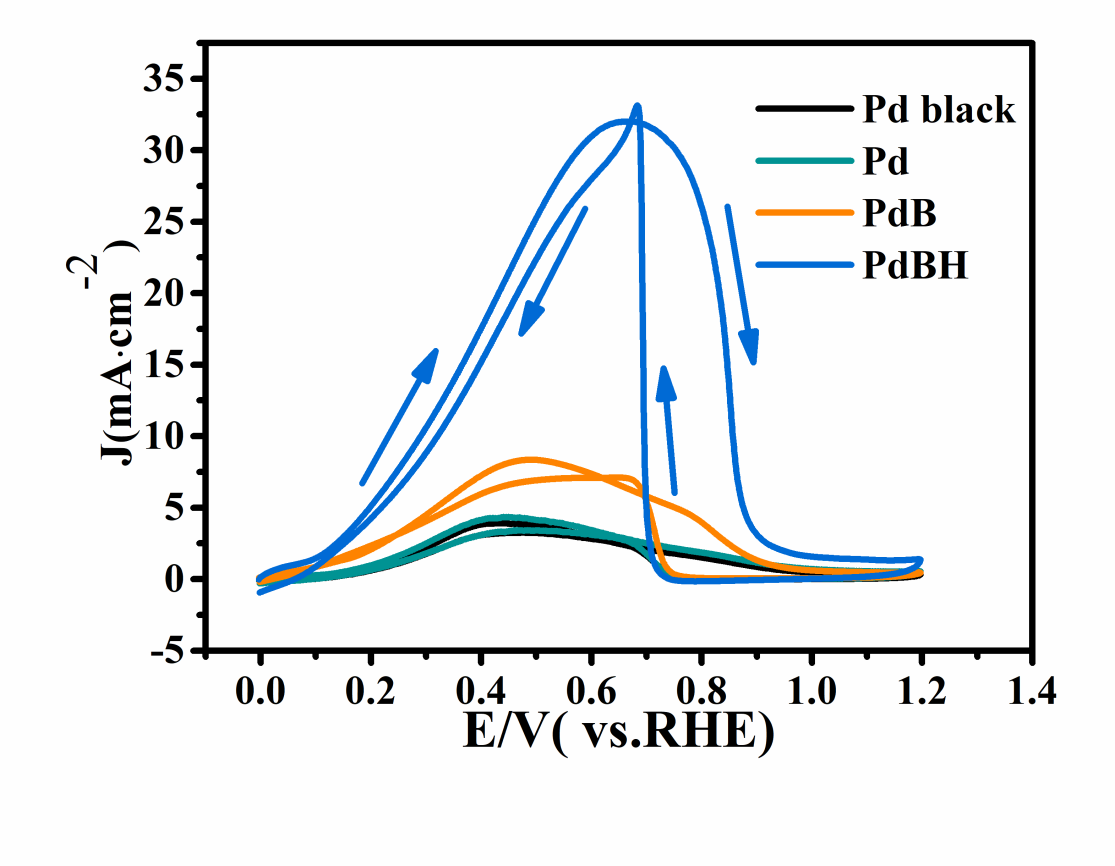


**Figure S14.** ECSA-normalized CV curves of the catalysts in a 0.5 M H_2_SO_4_ solution containing 0.5 M HCOOH.


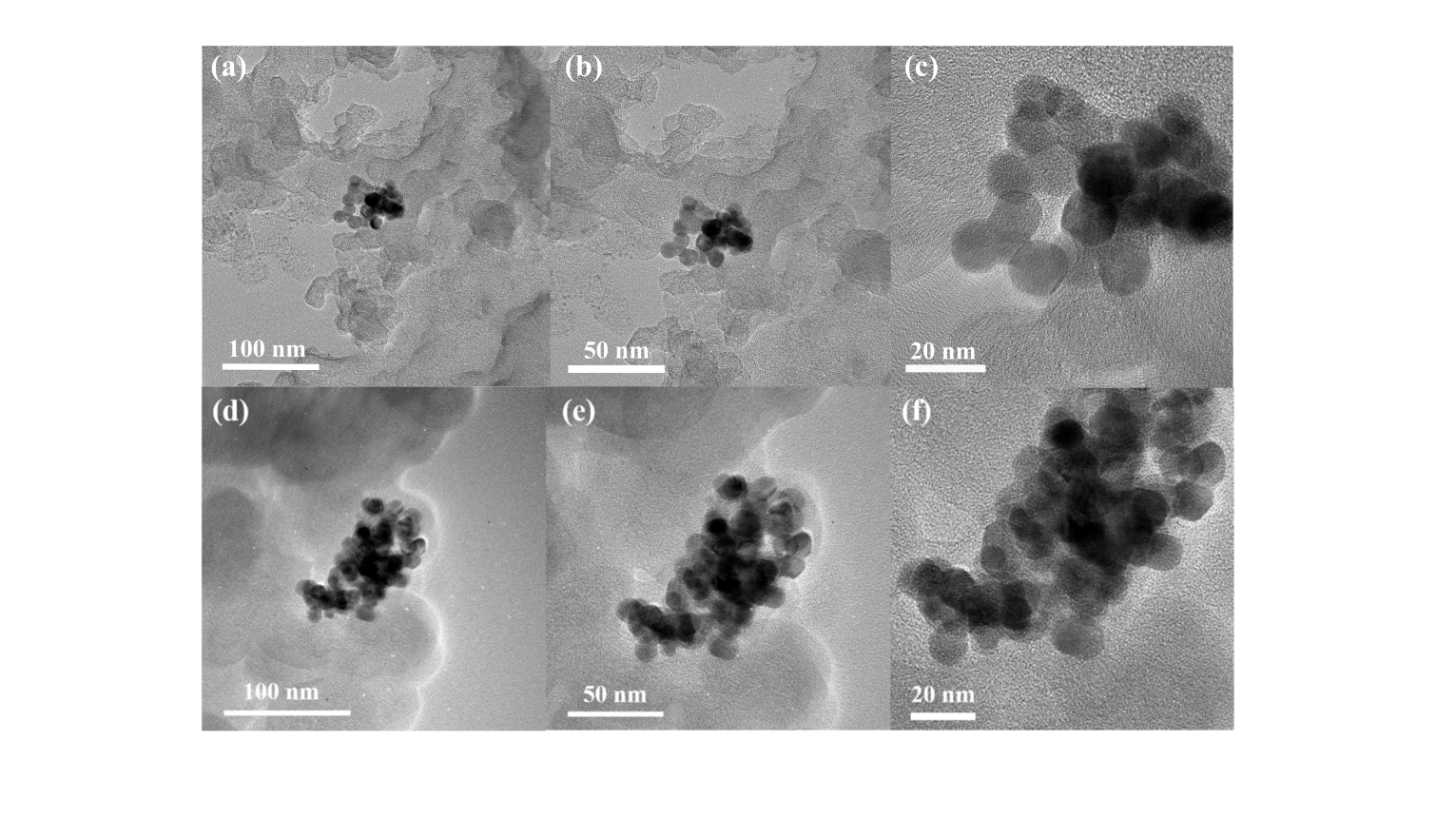


**Figure S15.** TEM images of PdBH/C obtained (a-c)before and (d-f)after 3600 s of chronoamperometric responses.


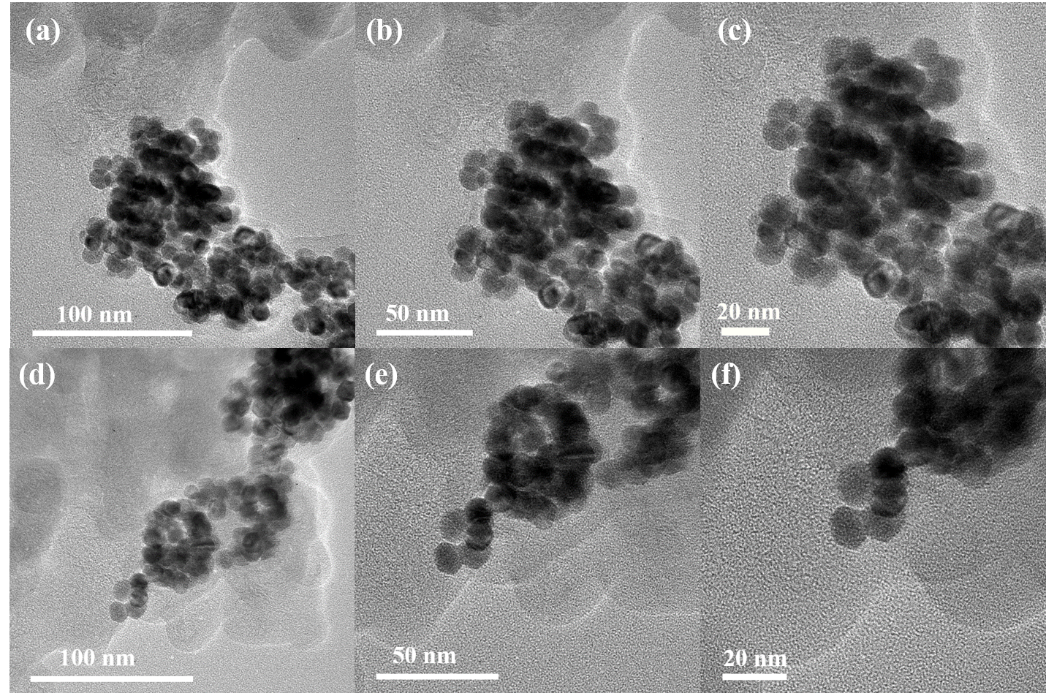


**Figure S16.** TEM images of PdB/C obtained (a-c)before and (d-f)after 3600 s of chronoamperometric responses.


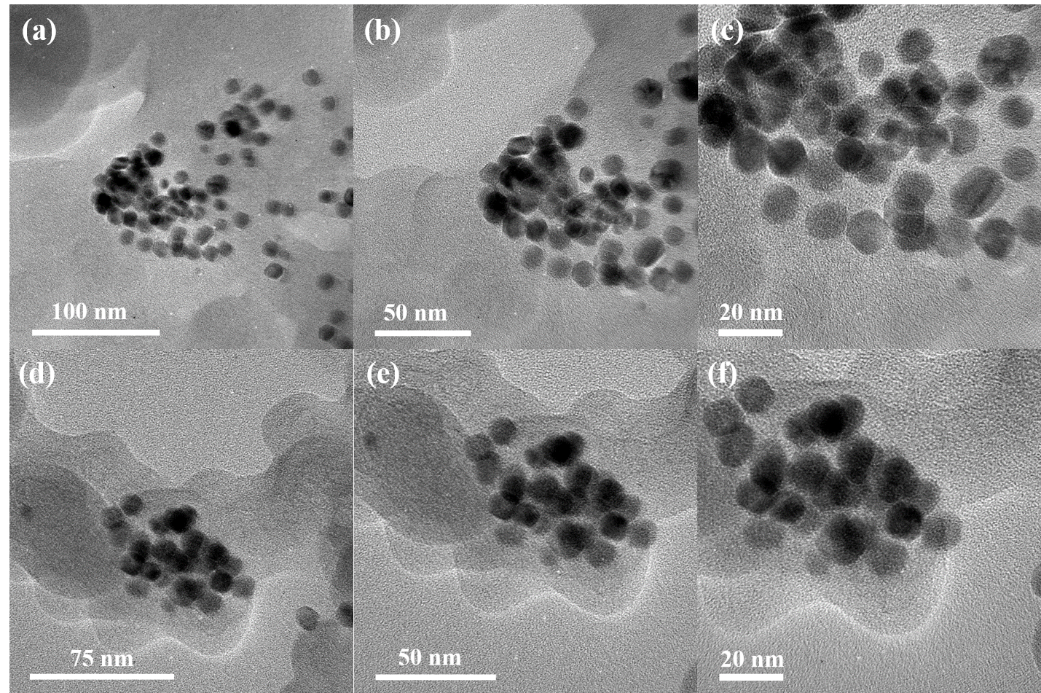


**Figure S17.** TEM images of Pd/C obtained (a-c)before and (d-f)after 3600 s of chronoamperometric responses.


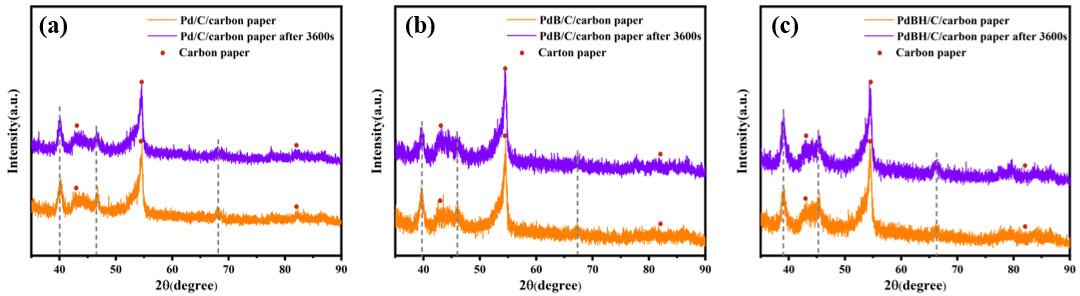


**Figure S18.** XRD patterns of (a) PdBH/C (b) PdB/C and (c) Pd/C obtained before and after 3600 s of chronoamperometric responses.


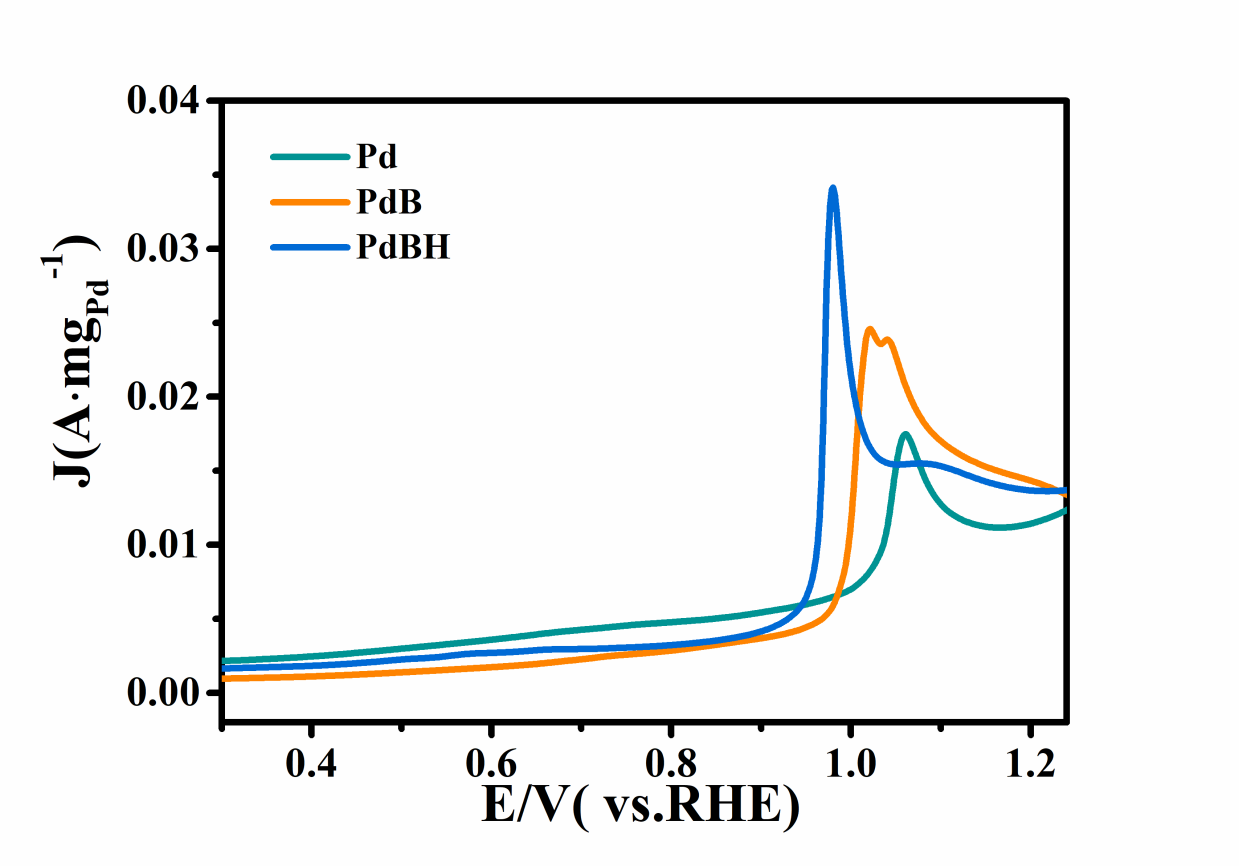


**Figure S19.** CO stripping curves of the Pd, PdB and PdBH catalysts in 0.5 M H_2_SO_4_ solution at a scan rate of 50 mV·s^-1^.


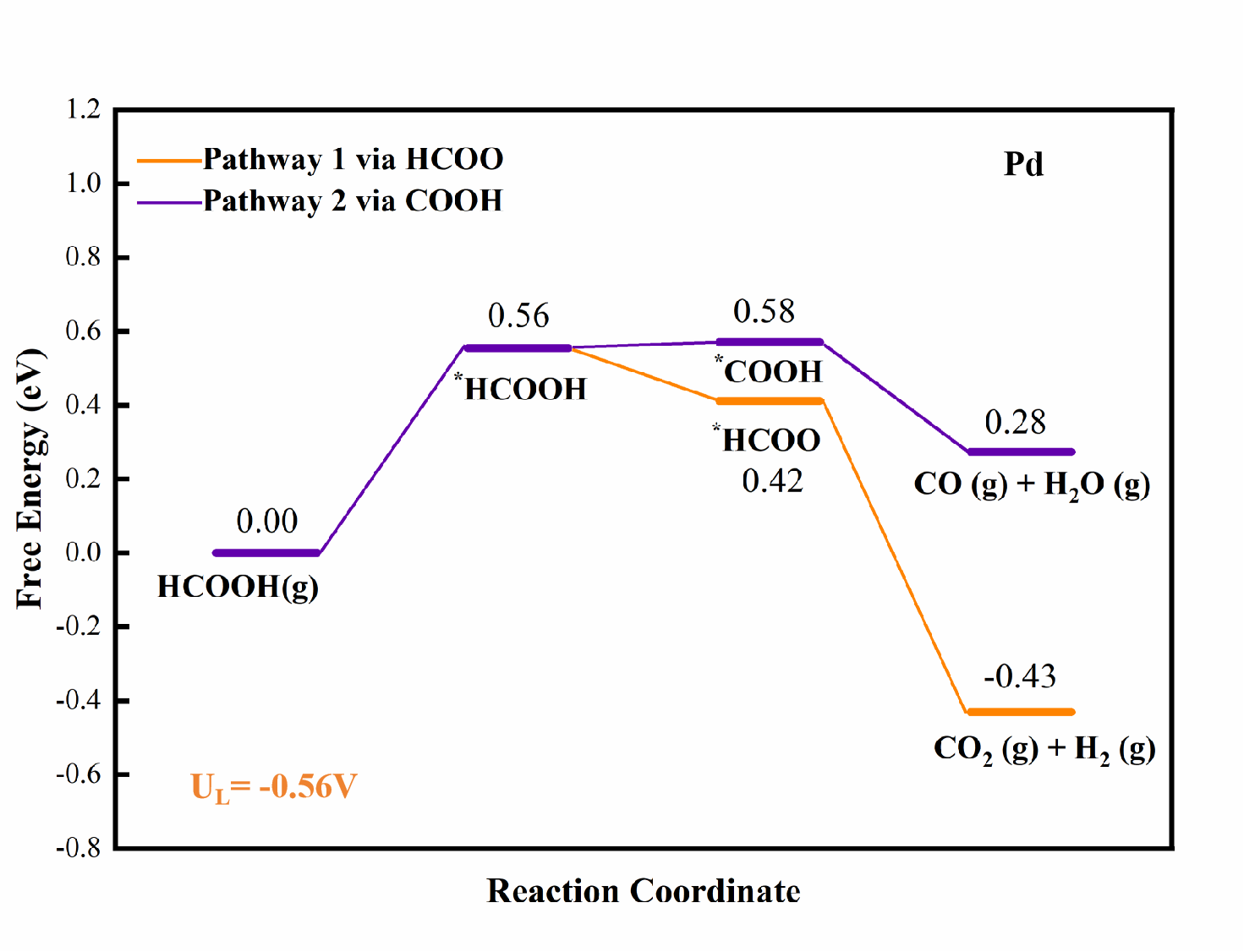


**Figure S20.** Comparison of the free energy profiles of Pd.


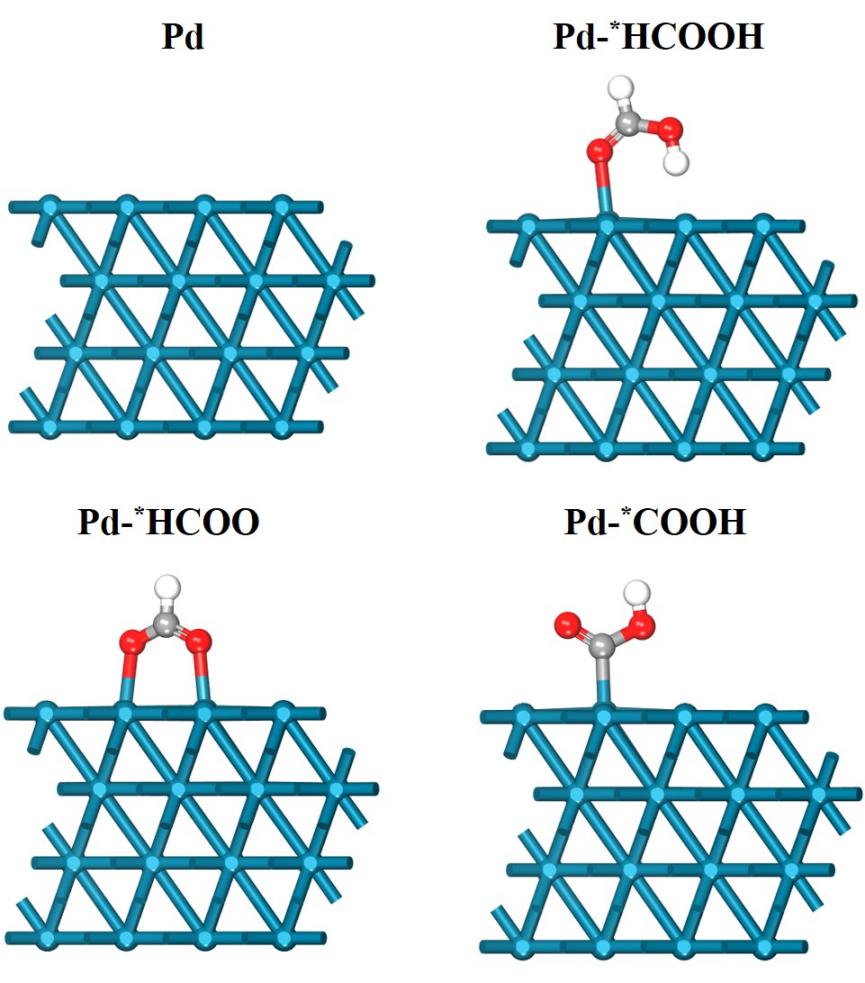


**Figure S21.** The optimized configurations of pure Pd (111) and adsorption configurations of ^*^HCOOH, ^*^HCOO, ^*^COOH intermediates on Pd (111) surfaces. The blue, red, grey, and white balls represent Pd, O, C, and H, respectively.


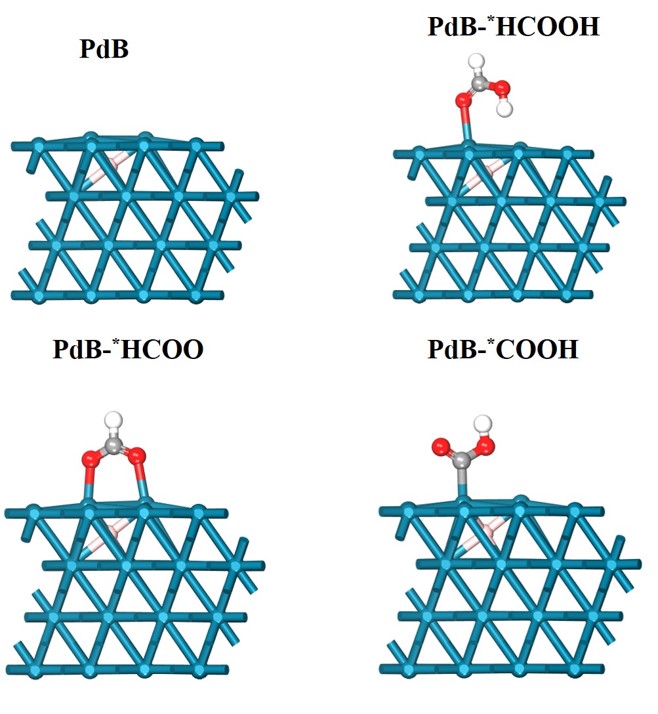


**Figure S22.** The optimized configurations of pure PdB (111) and adsorption configurations of ^*^HCOOH, ^*^HCOO, ^*^COOH intermediates on PdB (111) surfaces. The blue, pink, red, grey, and white balls represent Pd, B, O, C, and H, respectively.


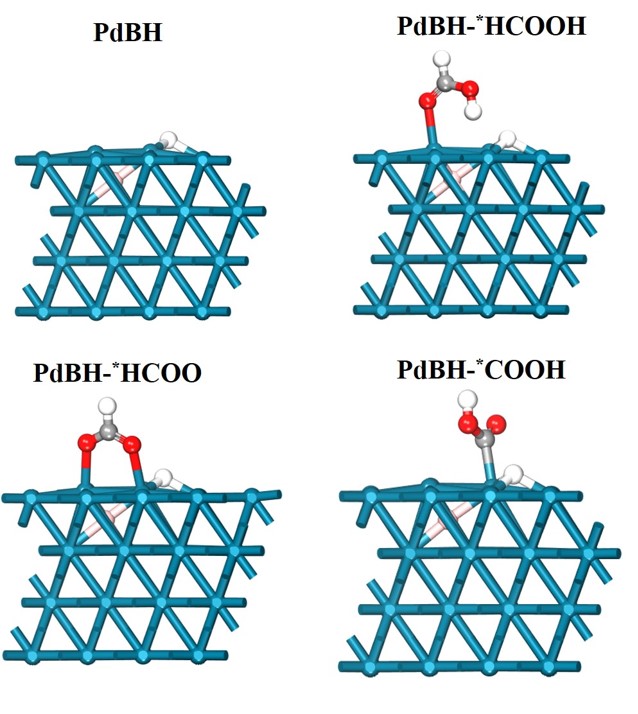


**Figure S23.** The optimized configurations of pure PdBH (111) and adsorption configurations of ^*^HCOOH, ^*^HCOO, ^*^COOH intermediates on PdBH (111) surfaces. The blue, pink, red, grey, and white balls represent Pd, B, O, C, and H, respectively.


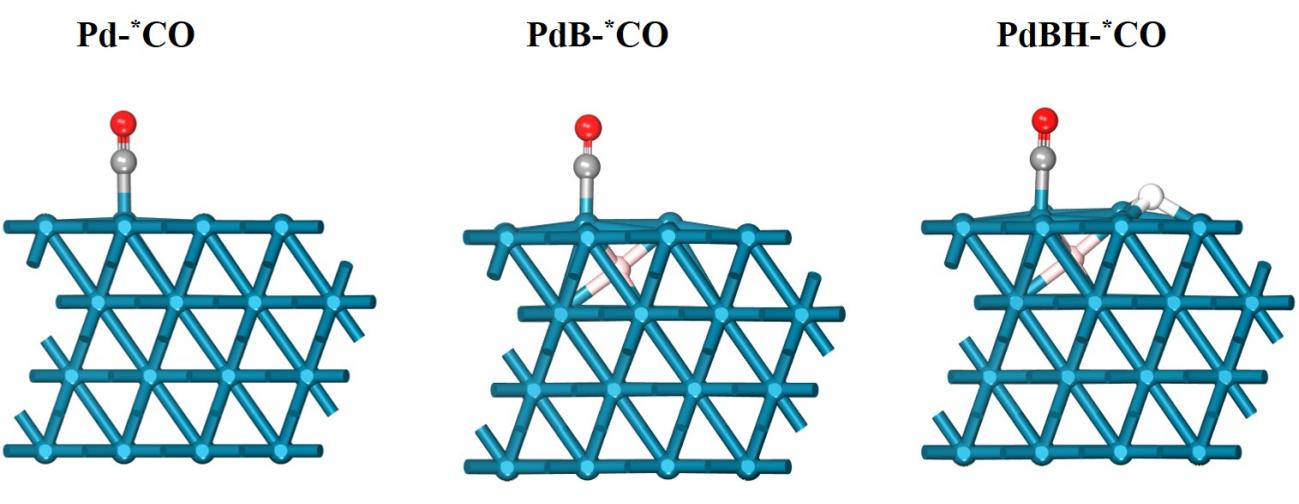


**Figure S24.** The optimized adsorption configurations of ^*^CO intermediates on Pd (111), PdB (111) and PdBH (111) surfaces. The blue, pink, red, grey, and white balls represent Pd, B, O, C, and H, respectively.

**Table S1. Structural parameters extracted from EXAFS fitting.**

| **Sample** | **Edge** | **Path** | **CN** | **R(Å)** | **σ^2^(10-^3^Å^2^)** | **ΔE_0_(eV)** | **R-factor** |
| --- | --- | --- | --- | --- | --- | --- | --- |
| Pd foil | Pd-K | Pd- Pd | 12.0 | 2.73 | 5.30 | 3.95 | 0.007 |
| PdB | Pd-K | Pd- B | 2.05 | 2.07 | 9.80 | -3.87 | 0.013 |
|  |  | Pd- Pd | 8.99 | 2.75 | 6.49 | -9.91 |  |
| PdBH | Pd-K | Pd- B | 1.53 | 2.07 | 3.74 | -7.38 | 0.008 |
|  |  | Pd- Pd | 8.23 | 2.76 | 7.97 | -5.19 |  |

Notes: CN, coordination number; R, distance between absorber and backscatter atoms. σ^2^, Debye-Waller factor; ΔE_0_, the inner potential difference between the reference compound and the experimental sample. R-factor, the goodness of fit. *S*0^2^ was fixed to 0.80, according to the experimental EXAFS fit of Pd foil by fixing *CN* as the known crystallographic value. Fitting conditions: *k* range：3.0 - 12.0; *R* range: 1.0-3.0; fitting space: R space; *k*-weight = 3. A reasonable range of EXAFS fitting parameters: 0.800 < *Ѕ*_0_^2^ < 1.000; *CN >* 0; *σ*^2^ > 0 Å^2^; |Δ*E*_0_| < 10 eV; *R* factor < 0.02.

**Table S2.** The peak positions of Pd 3d5/2 and B 1s in the XPS spectra of PdBH, PdB, and Pd

|  | **Pd 3d_5/2_** | **B 1s** |
| --- | --- | --- |
| Pd | 335.34 eV | 187.91 eV |
| PdB | 335.74 eV | 188.29 eV |
| PdBH | 335.60 eV | 188.25 eV |

**Table S3.ICP-MS test results of the Pd , PdB and PdBH nanocrystals.**

| **Sample** | **Pd(wt.%)** | **B(wt.%)** |
| --- | --- | --- |
| Pd | 55.1862 | 0 |
| PdB | 71.3079 | 0.623 |
| PdBH | 75.4263 | 0.4490 |

**Table S4**. Comparison of electrochemical FAOR performances of Pd-based catalysts in some previous representative works and this work.

| **Catalyst** | **Electrolyte** | **Mass activity** | **Specific activity** | **Reference** |
| --- | --- | --- | --- | --- |
| PdBH nanocrystals | 0.5M H_2_SO_4_+0.5M HCOOH | 1.26 A∙ mg^−1^_Pd_ | 31.99 mA∙cm^-2^ | **This work** |
| B-PdRu NPAs | 0.5M H_2_SO_4_+0.5M HCOOH | 1.09 A∙ mg^−1^_Pd_ | 2.76 mA∙cm^-2^ | ^[2]^ |
| B-PdCuAu NAs | 0.5M H_2_SO_4_+0.5M HCOOH | 1.21 A∙ mg^−1^_Pd_ | 2.29 mA∙cm^-2^ | ^[4]^ |
| DP-PdCu/C | 0.5M H_2_SO_4_+0.5M HCOOH | 0.55 A∙ mg^−1^_Pd_ | 1.91 mA∙cm^-2^ | ^[5]^ |
| Pd@SnO_2_-NSs/C | 1 M HClO_4_ + 0.5 M HCOOH | 4.96 A∙ mg^−1^_Pd_ | 21.695mA∙cm^-2^ | ^[6]^ |
| PdAg NSs | 0.5M H_2_SO_4_+0.5M HCOOH | 0.987A∙ mg^−1^_Pd_ | 5.01 mA∙cm^-2^ | ^[7]^ |
| PdeNi/C | 0.5M H_2_SO_4_+0.5M HCOOH | - | 19.83 mA∙cm^-2^ | ^[8]^ |
| Pd_62.5_Cu_37.5_/C | 0.5M H_2_SO_4_+0.5M HCOOH | 1.05 A∙ mg^−1^_Pd_ | - | ^[9]^ |
| PdH_0.43_-150℃ | 0.5M H_2_SO_4_+0.25M HCOOH | 1.06 A∙ mg^−1^_Pd_ | - | ^[10]^ |
| Pd_3_Au/C | 0.1M HClO_4_+ 0.1M HCOOH | 0.52 A∙ mg^−1^_Pd_ | 18.11 mA∙cm^-2^ | ^[11]^ |
| Pd nanosheet | 0.5M H_2_SO_4_+0.25M HCOOH | 0.41 A∙ mg^−1^_Pd_ | - | ^[12]^ |
| Pd−Sn−INNs | 0.5M H_2_SO_4_+0.25M HCOOH | 0.55 A∙ mg^−1^_Pd_ | 2.975 mA∙cm^-2^ | ^[13]^ |
| Cu_3_PdN NPs | 0.5M H_2_SO_4_+0.25M HCOOH | 0.87 A∙ mg^−1^_Pd_ | - | ^[14]^ |
| Pd/PG | 0.5M H_2_SO_4_+0.25M HCOOH | 1.05 A∙ mg^−1^_Pd_ | 84.7 mA∙cm^-2^ | ^[15]^ |
| 3D-RGO/Pd-NWs | 0.5M H_2_SO_4_+0.25M HCOOH | 0.97 A∙ mg^−1^_Pd_ | - | ^[16]^ |

# References

[1] M. Jin, H. Liu, H. Zhang, Z. Xie, J. Liu, Y. Xia, *Nano Research* **2010**, *4*, 83-91.

[2] S. Liu, Z. Wang, H. Zhang, S. Yin, Y. Xu, X. Li, L. Wang, H. Wang, *Nanoscale* **2020**, *12*, 19159-19164.

[3] aK. Zhang, C. Wang, F. Gao, S. Guo, Y. Zhang, X. Wang, S. Hata, Y. Shiraishi, Y. Du, *Coordination Chemistry Reviews* **2022**, *472*, 214775; bG. Wang, J. Liu, Y. Sui, M. Wang, L. Qiao, F. Du, B. Zou, *Journal of Materials Chemistry A* **2019**, *7*, 14876-14881.

[4] H. Wang, X. Qian, S. Liu, S. Yin, Y. Xu, X. Li, Z. Wang, L. Wang, *Chemistry – A European Journal* **2020**, *26*, 2493-2498.

[5] J. Geng, Z. Zhu, Y. Ni, H. Li, F. Cheng, F. Li, J. Chen, *Nano Research* **2021**, *15*, 280-284.

[6] Y.-W. Zhou, Y.-F. Chen, X. Qin, K. Jiang, W.-F. Lin, W.-B. Cai, *Journal of Catalysis* **2021**, *399*, 8-14.

[7] Z. Teng, M. Li, Z. Li, Z. Liu, G. Fu, Y. Tang, *Materials Today Energy* **2021**, *19*,100596

[8] X. Liu, Y. Bu, T. Cheng, W. Gao, Q. Jiang, *Electrochimica Acta* **2019**, *324*, 134816.

[9] J. Zheng, H. Zeng, C. Tan, T. Zhang, B. Zhao, W. Guo, H. Wang, Y. Sun, L. Jiang, *ACS Sustainable Chemistry & Engineering* **2019**, *7*, 15354-15360.

[10] J. Zhang, M. Chen, H. Li, Y. Li, J. Ye, Z. Cao, M. Fang, Q. Kuang, J. Zheng, Z. Xie, *Nano Energy* **2018**, *44*, 127-134.

[11] S.-Y. Lee, N. Jung, J. Cho, H.-Y. Park, J. Ryu, I. Jang, H.-J. Kim, E. Cho, Y.-H. Park, H. C. Ham, J. H. Jang, S. J. Yoo, *ACS Catalysis* **2014**, *4*, 2402-2408.

[12] X. Qiu, H. Zhang, P. Wu, F. Zhang, S. Wei, D. Sun, L. Xu, Y. Tang, *Advanced Functional Materials* **2017**, *27*.1603852

[13] D. Sun, L. Si, G. Fu, C. Liu, D. Sun, Y. Chen, Y. Tang, T. Lu, *Journal of Power Sources* **2015**, *280*, 141-146.

[14] J. Jia, M. Shao, G. Wang, W. Deng, Z. Wen, *Electrochemistry Communications* **2016**, *71*, 61-64.

[15] J. Zhao, Z. Liu, H. Li, W. Hu, C. Zhao, P. Zhao, D. Shi, *Langmuir* **2015**, *31*, 2576-2583.

[16] X. Qiu, P. Wu, L. Xu, Y. Tang, J. M. Lee, *Advanced Materials Interfaces* **2015**, *2(18),*1500321.

# Author Contributions

Huiling Li and Shangqi Zhou contributed equally to this work.
